# Moderating effects of coping on work stress and job performance for nurses in tertiary hospitals: a cross-sectional survey in China

**DOI:** 10.1186/s12913-017-2348-3

**Published:** 2017-06-12

**Authors:** Li Li, Hua Ai, Lei Gao, Hao Zhou, Xinyan Liu, Zhong Zhang, Tao Sun, Lihua Fan

**Affiliations:** 1 Department of Health Management, School of Public Health, Harbin Medical University, Baojian Road 157, Harbin, China; 2 Second Affiliated Hospital, Harbin Medical University, Harbin, China; 3 Department of Emergency, Harbin Center for Disease Control and Prevention, Harbin, China

**Keywords:** Coping, Nurses, Work stress, Job performance, Moderation

## Abstract

**Background:**

Work stress is a major problem for nurses and it can negatively influence job performance. Therefore, it is critical to explore variables that can reduce or buffer the negative effects of work stress. This study explores the moderating effects of coping strategies on the relationship between work stress and job performance for nurses in China.

**Methods:**

A cross-sectional survey of 852 nurses from four tertiary hospitals in Heilongjiang Province, China, was conducted in 2013. Descriptive statistics were reported for socioeconomic status and demographic characteristics, level of work stress, coping strategies, and job performance. Regression analysis was conducted to test the interaction between work stress and coping strategies on job performance.

**Results:**

Three subscales of work stress were negatively related to job performance. Positive coping strategies moderated Patient Care and job performance while negative coping strategies moderated Workload and Time and performance, and between Working Environment and Resources and performance.

**Conclusions:**

Positive coping strategies reduce or buffer the negative effects of work stress on job performance and negative coping strategies increased the negative effects.

## Background

Worldwide, nursing is known to be a very stressful occupation. Studies from China reported that nurses work under great pressure because of a heavy workload, contending with death and dying, inter-staff conflict, lack of resources, and insufficient training [1]. Long term stress may affect hospitals through nurses’ dissatisfaction, burnout, poor performance, or turnover intention [2–5]. Since nurses are the frontline staff of the healthcare team this can reduce the quality of health services. Therefore, it is important for both nurses and their managers to take measures to reduce work stress.

Work stress can be defined as a mismatch between an individual and their environment [6]. In general, the higher the imbalance between external demands and an individual’s abilities, the higher the level of stress that will be experienced [7].

In recent years, there has been a substantial amount of research on the relationship between work stress and job performance. Some of this research revealed that high work stress lead to low job performance [2]. However, a few studies found an inverted U-shaped relationship or a positive relationship between work stress and job performance [8, 9]. Wu et al. asserted that a possible explanation for these inconsistent results might be existing variables to moderate the effect of stress on performance [10]. A broad range of variables have been considered as potential moderators such as emotional intelligence, organizational commitment, and supportive leadership [11, 12]. Folkman’s study showed that effective coping strategies can avoid or reduce stress levels [13].

According to the Transactional Model of Stress, coping is an integral element in the stress process because coping strategies can help alleviate the effects of stressors on strains [14].

Previous studies have identified two main types of coping strategies: emotion-focused and problem-focused strategies [13]. Later analyses subdivided emotional-focused strategies into escape-avoidance, distancing, self-control, and positive reappraisal [15]. Other researchers have categorized coping strategies into positive or constructive coping and negative or destructive coping [16].

Recent research revealed that coping strategies played an important moderating role in work stress and well-being as well as job satisfaction among nurses and administrators [17, 18]. However, there continues to be a lack of information on the moderating effects of coping strategies on work stress and job performance for Chinese nurses.

Based on the Transactional Model of Stress, the aim of this study was to examine the moderating effects of coping strategies on work stress and job performance. Specifically, we explore: (a) the levels of work stress, coping strategies, and job performance that occurs in nursing; (b) the associations among stress, coping strategies, and job performance; and (c) whether coping strategies moderate the relationship between work stress and job performance.

## Methods

### Sample

A cross-sectional questionnaire survey was conducted in Heilongjiang Province, China, from September 2013 to December 2013. Two tertiary university-affiliated hospitals and two tertiary non-affiliated hospitals were selected. There were 8–10 types of clinical departments in these four hospitals. One sub-department was randomly selected in each kind of clinical department in each hospital. The data were collected anonymously and the participants completed the questionnaires privately to ensure confidentiality. The questionnaire included a cover page explaining the purposes and procedures of the study. Respondents were assured that participation in the survey was voluntary, and the return of questionnaires represented informed consent. Nurses who take part in the survey were able to choose the best time to complete the questionnaire. Most questionnaires were collected on site by the investigator on the day of the visit. If some nurses did not finish that day, investigators set a date to retrieve the questionnaires. The questionnaire was relatively brief and no private personal information was collected. In total, 1057 questionnaires were distributed and 1027 questionnaires were returned for a response rate of 97.2%. However, 175 were incomplete or even blank, which left 852 (80.6) valid questionnaires. This study was approved by Medical Ethic Committee of Harbin Medical University.

### Assessment tools

#### Job performance scale

In this study, we used a 5-item scale developed by Williams and Anderson to examine in-role job performance (e.g. “I effectively fulfilled my roles and responsibilities concerning the hospital’s proposal assignment”) [19, 20]. This self-report tool asked participants to rate their responses on a 7-point scale ranging from 1 (highly disagree) to 7 (highly agree). Then, scores were averaged across items to form a scale score. In terms of reliability, the Cronbach’s alpha of the task performance scale in the present study was 0.85. The result of correlation test showed that there was a significant correlation between the averaged scores and individual score of five items, which proved to be of good validity (correlation = 0.47–0.75).

#### Work stress scale

Taking into consideration the Chinese cultural background of participants and acceptable reliabilities of available instruments, work stress was measured with a 35-item scale developed by Li and Liu which named Chinese Nurse Job Stressors Questionnaire (e.g. “few opportunities for improvement”) [21]. This scale is composed of five subscales (i.e., Nursing Profession and Clinical Duty, Workload and Time, Working Environment and Resources, Patient Care, and Management and Interpersonal Relationship). Respondents were asked to indicate the strength of their agreement with the statements on a 4-point scale ranging from 1 (highly disagree) to 4 (highly agree). The scale achieved reasonable reliability in our sample, obtaining an overall Cronbach’s alpha of 0.92. In addition, the Cronbach’s alpha for the individual scales ranged from 0.81 to 0.88. Scores of 35items were averaged to form the general work stress scores. We identified the general work stress as criterion validity. The result of correlation test showed that there was a significant correlation between five subscales of work stress and the general work stress. (Correlation = 0.39–0.76).

#### Coping strategies scale

The 20-item version of the Simple Coping Style Scale, which was developed by Xie was used to examine coping strategies used by nurses [22]. The scale classified coping strategies into positive coping (12 items) (e.g. “looking for support from family or friends, utilizing others’ ways of dealing with similar problems”) and negative coping (8 items) (e.g. “procrastinating, relying on others, trying to forget about everything”). Respondents were asked to indicate the frequency that they used each strategy with a 4-point scale ranging from 0 (never used) to 3 (often used). In the present study, the scale demonstrated appropriate reliability and the Cronbach’s alpha was 0.86. Scores of 20 items were averaged to form a scale score. We identified the scale score as criterion validity. The result of correlation test showed that there was a significant correlation between positive coping strategies, negative coping strategies and the scale score. (Correlation = 0.29–0.74).

### Data analysis

In this study, survey results were analyzed using SPSS V.19.0. There were four main components to the data analysis. First, descriptive statistics were reported for socioeconomic and demographic status, work stress, coping strategies, and job performance. Second, correlations among work stress, coping strategies, and job performance were assessed using correlational analysis. Third, regression analysis was conducted to test potential moderating effects [23]. Centered scores were used for the interaction terms to avoid multicollinearity. Finally, significant interactions were explored by examining conditional effects with contrasts and plots, and simple effects tests were conducted to determine whether the slopes significantly differed from zero. A *p*-value of 0.05 was considered to have statistical significance.

## Results

### Description of respondents

Table 1 reports participants’ socioeconomic and demographic characteristics. Participants were predominantly female (96.6%) and had attained a bachelor’s degree (63.8%). Only 5.7% had a senior professional title. The average age was 28 years and 87.7% of respondents were under 35 years of age. About 80% had worked less than 10 years in their current position. In terms of marital status, 45.5% were married and 54.5% were single, separated, or divorced.

**Table 1:** Demographic characteristics of respondents (*N* = 852)

Variables		*N* (%)
Gender	Female	822 (96.5)
Age in years	<25	242 (28.4)
	25–34	502 (58.9)
	35–44	76 (8.9)
	≥45	32 (3.8)
Mean age	28.20 ± 6.85	
Marital status	Married	388 (45.5)
	Others	457 (54.5)
Educational level	Secondary technical certificate	20 (2.4)
	Associate degree	271 (31.8)
	Bachelor’s degree	544 (63.8)
	Master’s degree	17 (2.0)
Professional title	Nurse	393 (46.1)
	Nurse Practitioner	290 (34.0)
	Nurse-in-charge	121 (14.2)
	Associate professor of nursing	32 (3.8)
	Professor of nursing	16 (1.9)
Administrative position	Nursing assistant	24 (2.8)
	Clinical nurse	692 (81.3)
	Group leader nurse	77 (9.0)
	Head nurse	59 (6.9)
Monthly income (RMB)	≤2000	52 (6.1)
	2001–4000	453 (53.2)
	4001–6000	289 (33.9)
	6001–8000	41 (4.8)
	>8000	17 (2.0)
Tenure (years)	<5	471 (55.3)
	6–10	205 (24.1)
	11–15	82 (9.6)
	≥16	94 (11.0)

From Table 2 it can be seen that Nursing Profession and Clinical Duty (M = 3.17, SD = 0.62) emerged as the major stressor, followed by Workload and Time (M = 2.93, SD = 0.61), Working Environment and Resources (M = 2.67, SD = 0.77), Patient Care (M = 2.55, SD = 0.63), and Management and Interpersonal Relationship (M = 2.46, SD = 0.61). Mean scores of positive and negative coping strategies were 1.98 (SD = 0.55) and 1.37, respectively. Results also indicated the average level of job performance was 5.64 (SD = 0.59).

**Table 2:** Means, standard deviations (SD), and correlations among work stress, coping strategies and job performance

	Mean	SD	1	2	3	4	5	6	7
1. Management and Interpersonal Relationship	2.46	0.61							
2. Nursing Profession and Clinical duty	3.17	0.62	0.283*						
3. Workload and Time	2.93	0.61	0.474*	0.595*					
4. Patient Care	2.55	0.63	0.539*	0.227*	0.374*				
5. Working Environment and Resources	2.67	0.77	0.481*	0.348*	0.433*	0.369*			
6. positive coping	1.98	0.55	−0.211*	−0.089*	−0.180*	−0.131*	−0.089*		
7. negative coping	1.37	0.67	0.229*	0.080*	0.089*	0.218*	0.181*	0.153*	
8. job performance	5.64	0.59	−0.522*	−0.387*	−0.413*	−0.321*	−0.404*	0.279*	-0.142*

*P < 0.01

### Correlations among the study variables

The correlations among the study variables are presented in Table 2. Each subscale of work stress had a significant negative correlation with job performance and positive coping strategies (*r* = −0.09 to −0.52, *p* < 0.01) and a significant positive correlation with negative coping strategies (*r* = 0.09 ~ 0.23, *p* < 0.01). In addition, positive coping strategies were positively correlated with job performance (*r* = 0.28, *p* < 0.01) and negative coping strategies were negatively correlated with job performance (*r* = −0.14, *p* < 0.01).

### Testing direct effects and moderating effects

Table 3 demonstrates the three models used to test the direct effect and moderating effects of work stress and coping strategies on job performance. In Model 1, the inclusion of the five work stress subscales resulted in Nursing Profession and Clinical Duty, Workload and Time, and Management and Interpersonal Relationship as negative predictors of job performance (adjusted R^2^ = 0.36, *p* < 0.01). In Model 2, the inclusion of five work stress subscales and coping strategies resulted in three subscales of work stress (i.e. Nursing Profession and Clinical Duty, Workload and Time, and Management and Interpersonal Relationship). In addition, negative coping strategies were negative predictors, while positive coping strategies were positive predictors of job performance (adjusted R^2^ = 0.41, *P* < 0.01).

**Table 3:** Regression analysis for testing moderation effects of coping on work stress and job performance

Variables	Model 1		Model 2		Model 3	
	β	*p*	β	*p*	β	*p*
Subscales of work stress						
Nursing profession & clinical duty	−0.39	0.00	−0.32	0.00	−0.31	0.04
Workload & time	−0.19	0.00	−0.25	0.00	−0.51	0.00
Working environment & resources	−0.06	0.12	−0.06	0.11	0.34	0.04
Patient care	−0.00	0.97	0.03	0.44	−0.42	0.00
Management & interpersonal relationship	−0.12	0.00	−0.10	0.00	0.16	0.25
Subscales of coping strategies						
Positive coping strategies			0.24	0.00	0.22	0.00
Negative coping strategies			−0.12	0.00	−0.16	0.00
Subscales of work stress × coping strategies						
Nursing Profession and Clinical Duty × positive coping					−0.18	0.43
Workload and Time × positive coping					0.045	0.81
Working Environment and Resources × positive coping					−0.257	0.24
Patient care × positive coping					0.436	0.01
Management and Interpersonal Relationship × positive coping					−0.054	0.73
Nursing Profession and Clinical Duty × negative coping					0.150	0.43
Workload & Time × negative coping					−0.468	0.01
Working Environment and Resources × negative coping					−0.531	0.01
Patient Care × negative coping					0.319	0.07
Management and Interpersonal Relationship × negative coping					−0.327	0.06
Adj. R^2^	0.36*		0.41*		0.42*	
R^2^-change	0.36*		0.05*		0.02*	

*P < 0.01

Model 3 augmented Model 2 through inclusion of the interaction terms (Work Stress × Coping Strategies). We found that positive coping strategies moderated the relationship between Patient Care and job performance (*β* = 0.44, *P* < 0.01). Negative coping strategies moderated the relationship between Workload and Time and job performance (*β* = −0.47, *P* < 0.01), and the relationship between Working Environment and Resources and job performance (*β* = −0.53, *P* < 0.05).

The significant moderations were probed following the procedures recommended by Aiken and West [24]. The values of the moderators (positive coping strategies and negative coping strategies) were chosen 1 SD above and 1 SD below the mean to form simple regression equations. Simple regression lines were generated by entering the values of the latent benefit variable 1 SD above and 1 SD below the mean in the simple regression equation that was formed in the previous step. The interaction was then plotted for each of the significant interaction terms to demonstrate effect of coping strategies on the relationship between work stress and job performance.

**Fig. 1:**
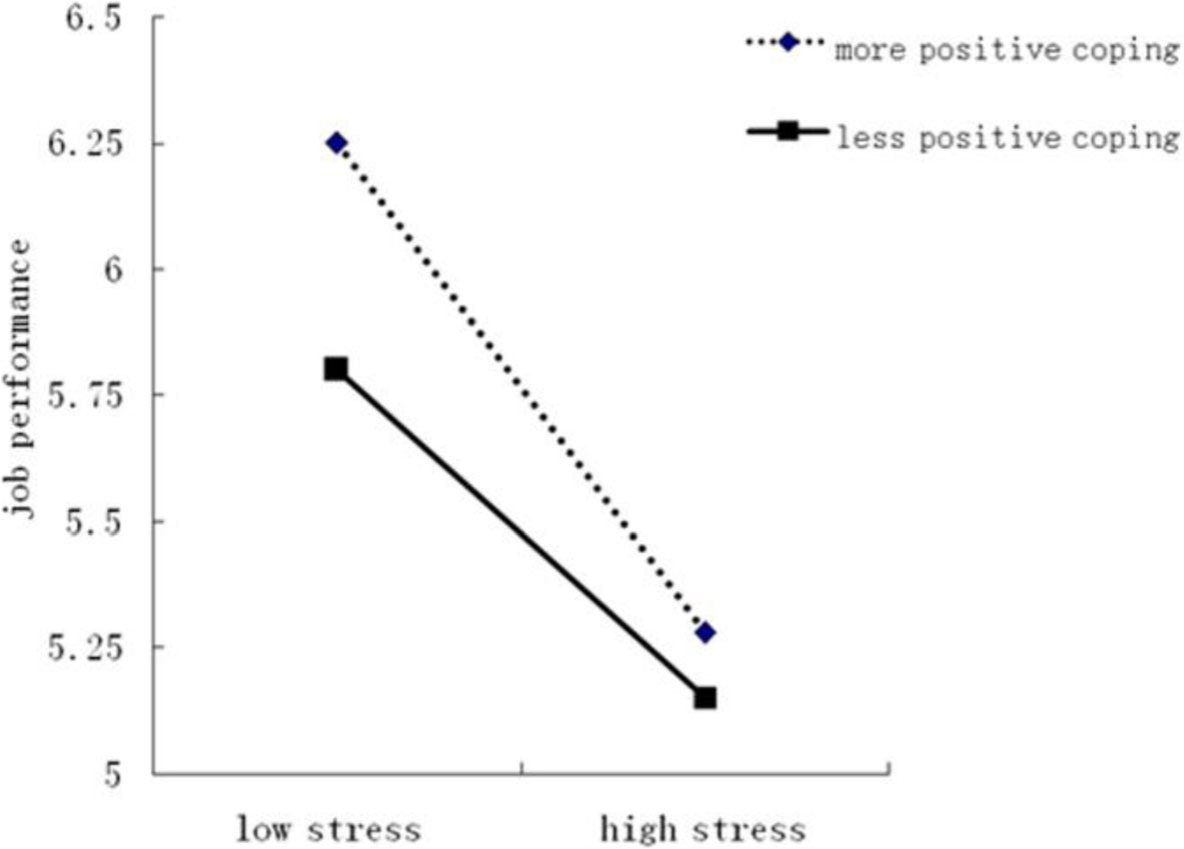
Moderation of positive coping on patient care stress and job performance

**Fig. 2:**
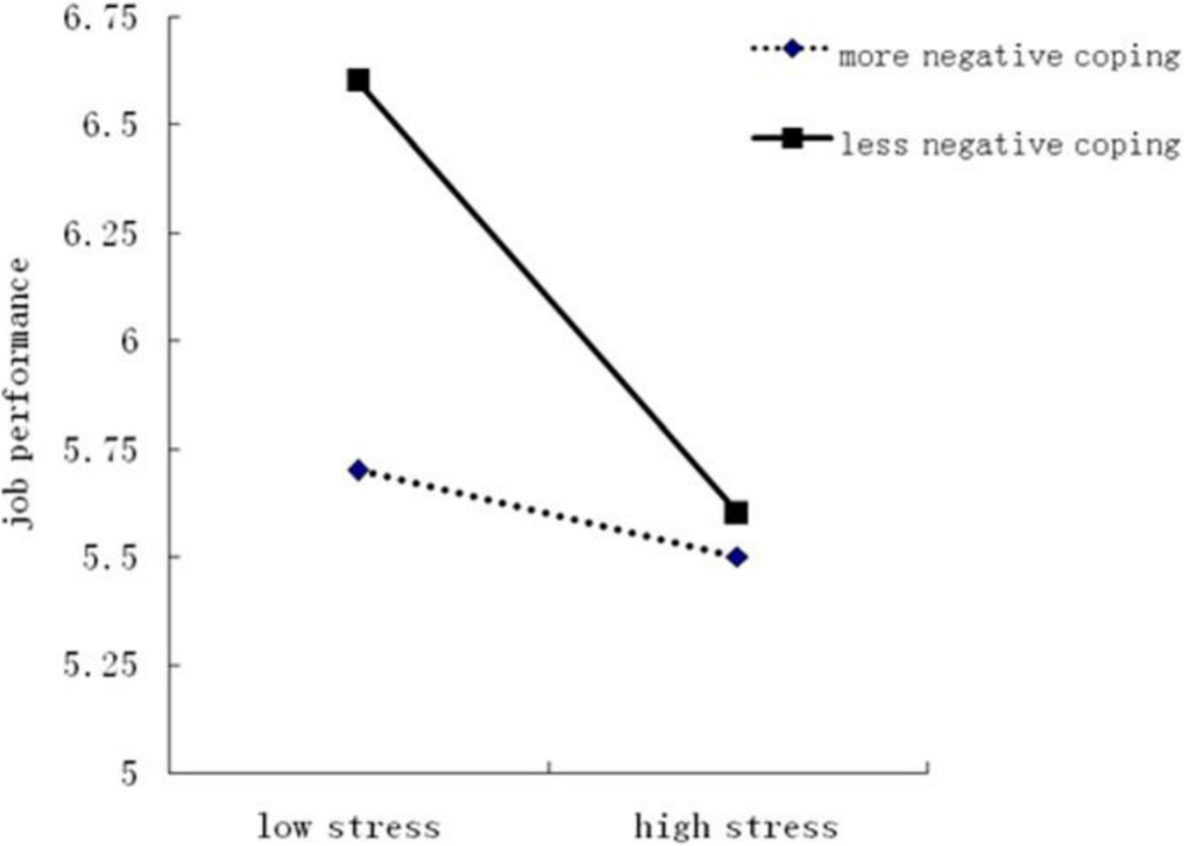
Moderation of negative coping on workload & time stress and job performance

**Fig. 3:**
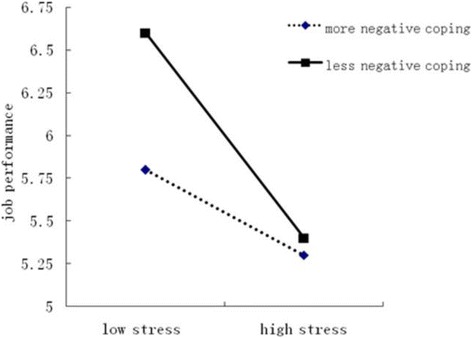
Moderation of negative coping on working environment & resources stress and job performance

Plots illustrating the significant moderations are displayed in Figs. 1, 2 and 3. Simple effects tests were conducted to determine whether the slopes significantly differed from zero (Aiken and West, 1991). The significant interaction term for Patient Care × positive coping strategies indicated that for both more positive coping strategies (*F* = 92.54, *p* < 0.01) and less positive coping strategies (*F* = 36.43, *p* < 0.01), increasing levels of work stress associated with decreasing levels of job performance. It also indicated that, with an increase in work stress, participants who used more positive coping strategies reported significantly higher job performance than those who used less positive coping strategies.

The significant interaction term for Workload and Time × negative coping strategies indicated that for less negative coping strategies, increasing levels of work stress were associated with decreasing levels of job performance (*F* = 67.49, *P* < 0.01) (Fig. 2). For more negative coping strategies, no association was found between levels of work stress and levels of job performance. The significant interaction term for Working Environment and Resources × negative coping strategies indicated that for both more negative strategies (*F* = 21.17, *P* < 0.01) and less negative strategies (*F* = 48.17, *P* < 0.01), increasing levels of work stress were associated with decreasing levels of job performance (Fig. 2). Furthermore, Figs. 2 and 3 indicated that with an increase in work stress, nurses who used less negative coping strategies reported significantly higher job performance than those who used more negative coping strategies.

## Discussion

The current study was one of the first of its kind to investigate the moderating effects of coping strategies on work stress and job performance among nurses in China. Results indicated that nurses used positive coping strategies more frequently than negative coping strategies. We also found that coping strategies had moderating effects on some of the work stress subscales and job performance.

In order to test the direct and moderating effects of coping strategies on work stress and job performance, a regression analysis was conducted (Table 3). First, when work stress variables were used (Model 1, Table 3), three subscales of work stress (i.e., Nursing Profession and Clinical Duty, Workload and Time, and Management and Interpersonal Relationship) had direct negative effects on job performance. These findings were consistent with previous studies that reported that excessive workload, lack of opportunities for promotion, and a lack of respect were major negative predictors of job performance [25].

Second, including negative predictors of job performance into the analysis produced a significant increase in the variance explained in job performance (Model 2, Table 3). This finding supports our expectation that coping strategies would help explain job performance. Consistent with the study by Lu et al., positive coping strategies were positively related to job performance and negative coping strategies were negatively related to job performance [26].

Furthermore, inclusion of the interaction terms (Stressors X Coping strategies) accounted for a larger proportion of the explained variance (Model 3, Table 3), indicating that coping strategies had a moderating effect. Specifically, we found that positive coping (e.g. looking for support from family or friends, utilizing others’ ways of dealing with similar problems) could reduce or buffer the negative effects of Patient Care on job performance. Negative coping strategies (e.g. procrastinating, relying on others, trying to forget about everything) can strengthen the negative effects of “Workload and Time” and “Working Environment & Resources” on job performance. Similarly, Atteya found that methods of coping with stress had an impact and exerted an influence on job performance [27]. Taken together; the present findings have significant implications for both managers and nurses in their efforts to improve job performance.

First, since work-related stress is negatively related to job performance, managers should pay more attention to the stress level of the nurses they work with. This study found that Nursing Profession and Clinical Duty was the highest perceived workplace stressor, followed by Workload and Time. Both of these factors were negatively related to job performance. Similarly, a substantial amount of existing research suggested that workload and professional and career issues were primary work stressors for nurses in China [23, 28–30]. There are several possible reasons for these findings. First, Chinese nurses have a relatively low social status and experience a lack of recognition by others. They often complain of verbal or physical violence by patients and their families. Second, compared to other health technical workers, nurses have few opportunities for promotion and further study [21]. Furthermore, excessive documentation and shortage of nurses increases nurses’ workload [31].

Second, nurses should adopt more positive coping strategies when faced with stress. This study revealed that positive coping strategies could reduce or buffer the negative effect of stress on job performance. Overall, respondents tended to use more positive than negative coping strategies contend with work stress. However, when work stress increased, the usage of positive coping strategies decreased and negative coping strategies increased (Table 2). Therefore organizations should create an environment that encourages workers to use more positive coping strategies when experiencing stress. The managers should investigate and analyze the causes and types of work stress, help nurses to recognize the stress and their own coping styles, and then make detailed stress-reduction plan for nurses on the level of organization. In the meanwhile, managers should carry out training about positive mood management, provide relevant information, and knowledge on stress management for nurses. Besides that, organizations can let nurses think their work is meaningful and beneficial work by developing scientific evaluation system based on key performance indicators.

Thirdly, coping strategies can only partly moderate the effects of work stress on job performance. Furthermore, the moderating effects on job performance were smaller under high stress than under low stress. Consequently, managers should take multiple measures to help nurses to reduce their work stress and in turn to improve the job performance such as creating a safe working environment, establishing adequate infrastructure and other resources, allocating a reasonable workload, and providing support when nurses experience challenges.

## Conclusion

This study was a preliminary attempt to explore the relationship between work stress, coping strategies, and job performance for nurses in China. Results of this study indicate that positive coping strategies moderated the relationship between Patient Care and job performance, while negative coping strategies moderated the relationship between Workload and time and job performance as well as between Working environment and resources and performance. As coping strategies can only moderate the effects of some subscales of work stress on job performance, managers should utilize multiple measures to help nurses to reduce work stress.

### Limitations

Three limitations of this study should be noted. First, performance was obtained from self-reports. Respondents may have underestimated or overestimated the level of their own performance. As we know, job performance can be assessed by objective indicators or subjective indicators. Different hospitals may have different performance evaluation index system, so it is difficult to assess job performance by objective indicators. Subjective assess can be reported by supervisors, colleagues and oneself. Job performance obtained from supervisors or colleagues may also reflect bias in reporting and the anticipated cost is high. So we use self-rated performance take into account the data availability, the accuracy and the cost. We recognized that this self- administration bias might have affected the results. Second, we used a cross-sectional survey, which may limit our ability to identify causal relationships between work stress and job performance. Thirdly, this study was based on a small sample of nurses in tertiary hospitals, which may limit the generalisability of the research findings. University-affiliated hospitals and non-affiliated hospitals may take on different tasks and workload, so a multistage, stratified sampling design was employed to ensure that study data were representative. In this study, two tertiary and two tertiary non-affiliated hospitals were selected. There were 8–10 types of clinical departments in these four hospitals. One sub-department was randomly selected in each kind of clinical department in each hospital.
